# Freshwater Macrophytes: A Potential Source of Minerals and Fatty Acids for Fish, Poultry, and Livestock

**DOI:** 10.3389/fnut.2022.869425

**Published:** 2022-04-11

**Authors:** Guddu Kumar, JaiGopal Sharma, Ravi Kumar Goswami, Avanish Kumar Shrivastav, Douglas R. Tocher, Neelesh Kumar, Rina Chakrabarti

**Affiliations:** ^1^Aqua Research Lab, Department of Zoology, University of Delhi, New Delhi, India; ^2^Department of Biotechnology, Delhi Technological University, New Delhi, India; ^3^Institute of Aquaculture, Faculty of Natural Sciences, University of Stirling, Stirling, United Kingdom; ^4^Guangdong Provincial Key Laboratory of Marine Biotechnology, Shantou University, Shantou, China

**Keywords:** alpha-linolenic acid, linoleic acid, n-3 polyunsaturated fatty acids, sodium, iron, freshwater macrophytes

## Abstract

The freshwater macrophytes are abundant in tropical and subtropical climates. These macrophytes may be used as feed ingredients for fish and other animals. The nutritional value of twelve freshwater-cultured macrophytes was evaluated in the present study. Significantly higher crude protein (36.94–36.65%) and lipid (8.13–7.62%) were found in *Lemna minor* and *Spirodela polyrhiza*; ash content was significantly higher in *Hydrilla verticillata, Wolffia globosa*, and *Pistia stratiotes* (20.69–21.00%) compared with others. The highest levels of sodium, magnesium, chromium, and iron levels were recorded in *P. stratiotes*. *H. verticillata* was a rich source of copper, manganese, cobalt, and zinc; the contents of calcium, magnesium, strontium, and nickel were highest in *S. polyrhiza*. Selenium and potassium contents were higher in *Salvinia natans* and *W. globosa*, respectively. The n-6 and n-3 polyunsaturated fatty acids (PUFAs) contents were significantly higher in *W. globosa* and *Ipomoea aquatica*, respectively compared with others. Linoleic and α-linolenic acids were dominant n-6 and n-3 PUFAs. The highest value (4.04) of n-3/n-6 was found in *I. aquatica*. The ratio ranged from 0.61 to 2.46 in other macrophytes. This study reveals that macrophytes are rich sources of minerals, n-6 and n-3 PUFAs.

## Introduction

Freshwater macrophytes, the fastest growing aquatic plants, are abundant in tropical and subtropical countries. They grow profusely in nutrient-rich water. These macrophytes are broadly classified into four groups based on their occurrence in the water body: the surface floating (e. g. *Azolla* spp.), submerged (e. g. *Hydrilla* spp.), emergent (e. g. *Potamogeton* spp.), and marginal (e. g. *Ipomoea* spp.). The nutritional value of freshwater macrophytes has been recognized globally. The unchecked propagation of freshwater macrophytes creates problems in many water bodies. The judicial exploitation of these nutrient-rich plants may open a new avenue from a nutritional view point for humans and animals. The leaf protein extracted from freshwater macrophytes may be used for human or non-ruminant animals (1). Macrophytes are a rich source of protein, lipid, amino acids, fatty acids, and minerals (2). The amino acid and fatty acid profiles of duckweeds *Lemna minor* and *Spirodela polyrhiza* have been documented recently (3, 4). The mineral composition of macrophytes is different from the usual terrestrial vegetation. Calcium (Ca), iron (Fe), and manganese (Mn) contents are higher in aquatic plants compared with the terrestrial ones (1). Minerals are important catalysts for various biochemical reactions. These are essential components for metabolism, growth, and development and help the animals to cope with the variable environmental conditions (5). There is an optimum dose for each mineral. Low/high concentrations may affect the physiology of the organisms. Toxic minerals like arsenic (As), mercury (Hg), antimony (Sb), cadmium (Cd) etc., are required by the body in little amounts, whereas excess levels of useful minerals like, sodium (Na), potassium (K), magnesium (Mg), Ca, Fe etc., may be harmful (5). Dietary inclusions of polyunsaturated fatty acids (PUFAs) have several health benefits for humans and other animals. The study of the profiles of fatty acids of feed ingredients ensures the quality of diets. Fish are unable to synthesize two essential fatty acids like n-6 (derived from linoleic acid, LA) and n-3 (derived from alpha-linolenic acid, ALA). So these fatty acids should be supplied to the diets of fishes (6).

The evaluation of minerals and fatty acids' compositions of aquatic macrophytes is essential for their selection as potential feed ingredients for fish and other animals. Some of the commonly occurring freshwater macrophytes are: *Azolla microphylla, A. pinnata, Enhydra fluctuans, Hydrilla verticillata, Ipomoea aquatica, Lemna minor, Marsilea quadrifolia, Pistia stratiotes, Salvinia molesta, S. natans, Spirodela polyrhiza*, and *Wolffia globosa*. These macrophytes are distributed throughout the temperate, sub-tropical, and tropical regions of the world. Some of these macrophytes like, *E. fluctuans, I. aquatica*, and *M. quadrifolia*, are consumed as vegetables by humans in India and Bangladesh (7), and *W. arrhiza* has been consumed in Thailand (8). Most of these macrophytes, except *H. verticillata* (submerged plant), *M. quadrifolia*, and *E. fluctuans* (marginal plants) are surface floating macrophytes. All these macrophytes propagate through vegetative reproduction. Mosquito fern *Azolla* spp. (Azollaceae) are heterosporous free-floating ferns. It lives symbiotically with nitrogen-fixing blue-green algae *Anabaena azollae*. Watercress *Enhydra fluctuans* (Asteraceae) is a hydrophytic plant and it grows in canals and marshy places. Waterthyme *Hydrilla verticillata* (Hydrocharitaceae) is a submerged, rooted aquatic plant. It can grow in water up to a depth of 6 m, and in transparent water it can survive up to a depth of 12 m. The water spinach *Ipomoea aquatica* (Convolvulaceae) with hollow roots floats in water easily. Three members of the family Lemnaceae, namely *Lemna* spp., *Spirodela* spp., and *Wolffia* spp. are known as duckweeds. The plant consists of a single leaf or frond with one or more roots. Water clover *Marsilea quadrifolia* (Marsileaceae) is a deciduous, aquatic fern. Each green and thin stalk rises from the rhizome to the water surface; it contains a single shamrock-like leaf with four leaflets. Water cabbage *Pistia stratiotes* (Araceae) is a perennial monocotyledon with thick, soft, and light green leaves that form a rosette. It floats on the surface of the water and roots are hanging beneath the leaves. The short stolon connects both the mother and daughter plants. Water fern *Salvinia* spp. (Salviniaceae) is a perennial free-floating macrophyte. During the period of high growth, leaf size decreases and both leaves and stems fold, doubling and layering to cover more of the water surface. The nutritional value of macrophytes in terms of proteins, lipids, ash etc. varies greatly (2). The culture medium influences the mineral contents of the macrophytes (8). The extracts of seven freshwater macrophytes show no cytotoxic and anti-proliferative effects on human cell lines (9). Therefore, macrophytes should be considered as useful feed ingredients. Production of macrophytes using a standard technique may help to maintain the nutritional value of the plant and also maximize the health benefits.

The aim of the present study is to evaluate the nutritional value, *viz*. proximate composition, minerals and fatty acids profiles of twelve cultured freshwater macrophytes. This study will help to evaluate the suitability of these macrophytes as feed ingredients for fish, poultry, and livestock.

## Materials and Methods

### Culture of Macrophytes

Freshwater macrophytes were collected from water bodies of Delhi, Uttar Pradesh, and West Bengal and then identified. Macrophytes were cultured in outdoor cemented tanks (1.2 × 0.35 m) with clean dechlorinated tap water (3). A 10-cm layer of soil was used for the culture of *H. verticillata, M. quadrifolia*, and *E. fluctuans*. All other macrophytes were cultured without soil base. The depth of water was 30 cm in all culture tanks. A combination of organic manures *viz*. cattle manure, poultry droppings, and mustard oil cake was used (1:1:1) at the rate of 1.052 kg/m^3^. All manures were decomposed for 5 days and then macrophytes were introduced individually in the outdoor cemented tanks. Three replicates were used for each macrophyte. For the steady supply of nutrients for the growth of macrophytes, the same combination of manures (at one-fourth dose of the initial one) was applied in the culture tanks. Manures were decomposed for 5 days in separate containers and then applied on day 6. This schedule was followed throughout the culture period. Culture tanks were monitored regularly and macrophytes were harvested when the whole surface of the tank was covered with plants. The freshly harvested macrophytes were washed twice with tap water and then with distilled water. After air drying, macrophytes were kept at 40°C for 3 h. Then the ground, sieved, and fine powders were kept in air-tight containers and stored in a refrigerator at 4°C for further assay.

### Proximate Composition Analysis

The proximate composition of the macrophytes was analyzed (10). Three replicates were used for each assay. Moisture content was estimated after drying the sample at 105°C for 24 h. The dried samples were kept in a muffle furnace at 550°C for 8 h for the determination of ash contents. The crude protein contents were analyzed by measuring the nitrogen content (N x 6.25) with an automated micro-Kjeldhal apparatus (Pelican Instruments, Chennai, India). Crude lipid contents of the macrophytes were assayed gravimetrically (11). Carbohydrate contents were estimated by the subtraction method.

### Mineral Assay

The mineral compositions of macrophytes were assayed using Inductively-Coupled Plasma Mass Spectrometer (ICP-MS, Agilent 7900, USA) following standard protocol at the Instrumentation Facility of Indian Institute of Technology, New Delhi. The powdered macrophyte sample (150 mg) was taken in a closed digestion vessel and 8 ml of suprapure 69% nitric acid (HNO_3_, Merck, USA) was added to this. The sample was digested in Microwave digestion system (Multiwave PRO; Anton Paar, Austria). The digested sample was cooled at room temperature and transferred into a measuring cylinder; Milli-Q ultrapure water was added to make the volume 40 ml. Then the sample was filtered through a 0.2 μM syringe filter (Thermo Scientific, USA) and was collected in a glass vial. A 20 μL sample was injected through autosampler in the ICP-MS. The standard solution for each mineral was supplied with the equipment (Agilent Technologies, USA). It was diluted with Milli-Q ultrapure water containing 1% HNO_3_ to make concentrations of 20, 40, 60, 80, 100, 250, 500, 1000 μg/l. The calibration (standard) curve was prepared. The blank was prepared with Milli-Q ultrapure water containing HNO_3_ (1%). Minerals are divided into three major groups based on their concentrations in the mammal/human body *viz*. macro, trace, and ultra-trace minerals (5).

### Fatty Acid Analysis

The fatty acid profiles of the macrophytes were analyzed using Gas Chromatograph (GC)–Flame Ionization Detector, Clarus 580 (Perkin Elmer, USA). The total lipid extracted from plants (11) was used to prepare fatty acid methyl esters (FAME) by transesterification using sulfuric acid in methanol at 50°C for 16 h (12). After extraction and purification of FAME (13), 1 ml sample was kept in a glass vial of autosampler of GC. The sample was separated and quantified in a GC column (60 m × 0.32 mm i.d. × 0.25 μm ZB-wax, Phenomenex, UK). The data were collected from pre-installed program software (TotalChrom Workstation Ver6.3, Perkin Elmer). The FAME was identified with the help of standards (Supelco FAME 37 mix, Sigma-Aldrich, USA).

### Statistical Analysis

The compositions of twelve macrophytes are given as means ± standard error (SE). The differences in nutritional values of various macrophytes were tested using one-way analysis of variance (ANOVA) and Duncan's multiple range test (14). Statistical analyses were performed using the SPSS program (version 25.0). Statistical significance was accepted at *p* < 0.05.

## Results

### Proximate Composition

**Figure 1:**
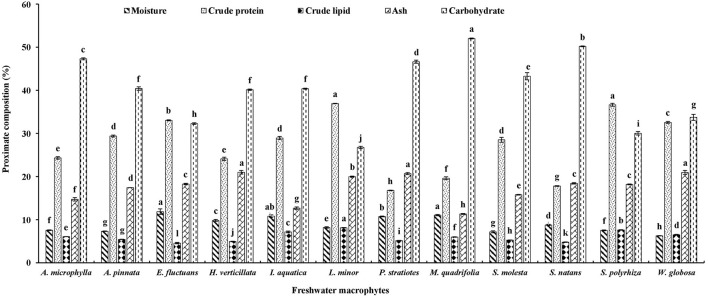
Proximate composition of twelve freshwater macrophytes cultured with organic manures. Bars with different superscripts are significantly different (*n* = 3).

The moisture content was highest (11.86%) and lowest (6.26%) in *E. fluctuans* and *W. globosa*, respectively (Figure 1). Significantly higher crude protein contents were found in two duckweeds, namely *L. minor* and *S. polyrhiza*, compared with others. The highest lipid content was also recorded in *L. minor*, followed by *S. polyrhiza*. The lipid content was minimum in *E. fluctuans*. Ash content was significantly higher in *H. verticillata, W. globose*, and *P. stratiotes* compared with other macrophytes. The ash content was minimum in *M. quadrifolia*. Carbohydrates levels were minimum and maximum in *L. minor* and *M. quadrifolia*, respectively.

### Mineral Composition

#### Macrominerals

**Figure 2:**
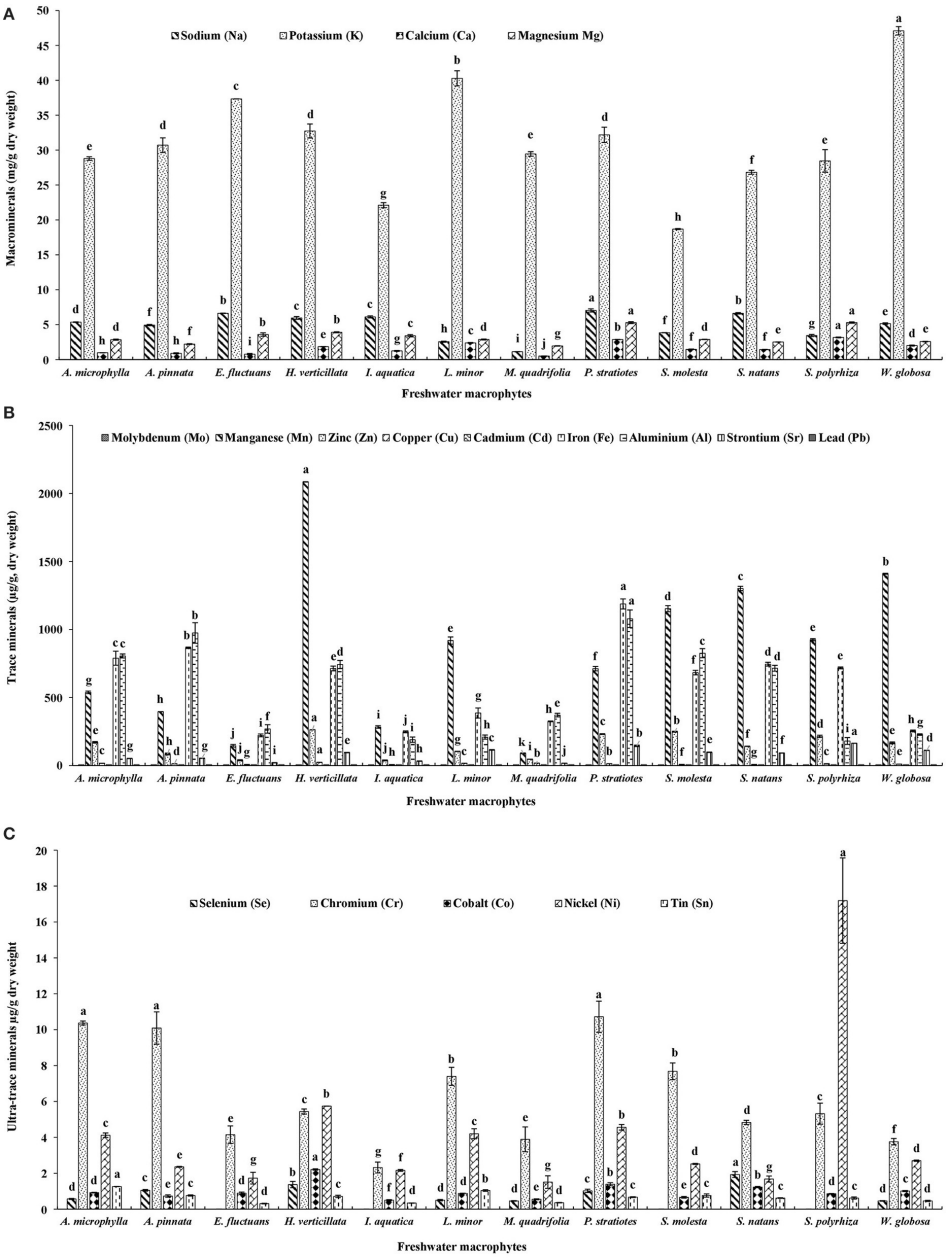
**(A)** Macrominerals, **(B)** trace minerals, and **(C)** ultra-trace minerals contents of twelve freshwater macrophytes cultured with organic manures. Bars with different superscripts are significantly different (*n* = 3).

Among these twelve freshwater macrophytes, Na content was significantly higher in *P. stratiotes* compared with others (Figure 2A). This group was followed by *S. natans* and *E. fluctuans*. A significantly higher K level was found in *W. globosa* compared with others. This was followed by *L. minor, E. fluctuans, H. verticillate*, and *P. stratiotes*. Ca content was highest in *S. polyrhiza*, followed by *P. stratiotes*. A significantly higher Mg level was found in *P. stratiotes* and *S. polyrhiza* compared with others. The Na, Ca, and Mg contents were minimum in *M. quadrifolia* compared with other macrophytes. It indicates the nutritional value of the macrophytes.

#### Trace Minerals

A total of nine trace minerals were found in these macrophytes (Figure 2B). Molybdenum (Mo) content was significantly higher in *A. microphylla, A. pinnata*, and *P. stratiotes* compared with others. Mn, zinc (Zn), copper (Cu), and Cd contents were significantly higher in *H. verticillata* compared with others. In *P. stratiotes*, significantly higher levels of Fe and aluminum (Al) were found compared with others. Among these macrophytes, *A. pinnata* ranked second for both Fe and Al. *A. microphylla* ranked third for Fe and fourth for Al contents. Maximum strontium (Sr) level was recorded in *S. polyrhiza* followed by *P. stratiotes*. In all these macrophytes lead (Pb) was found.

#### Ultra-Trace Minerals

Five ultra-trace minerals were found in these macrophytes (Figure 2C). A significantly higher level of selenium (Se) was found in *S. natans* compared with others. This plant was followed by *H. verticillata* and *P. stratiotes*. Se was absent in *S. molesta, E. fluctuans, I. aquatica*, and *S. polyrhiza*. Chromium (Cr) content was significantly higher in *P. stratiotes, A. microphylla*, and *A. pinnata* compared with others. Cobalt (Co) content was significantly higher in *H. verticillata* compared with others. This was followed by *P. stratiotes* and *S. natans*. Nickel (Ni) and tin (Sn) levels were significantly higher in *S. polyrhiza* and *A. microphylla*, respectively compared with others. Among these macrophytes, *P. stratiotes* ranked third for Ni content.

### Fatty Acid Profile

The fatty acid profiles of twelve freshwater macrophytes were documented in the present study (Tables 1–3). The saturated fatty acids (SFAs) content was significantly higher in *W. globosa* compared with others. This was followed by *A. pinnata, L. minor*, and *I. aquatica*. SFA content was minimum in *P. stratiotes*. Among, SFA, palmitic acid (C16:0) was the dominant one in all these plants. Monounsaturated fatty acids (MUFA) content was significantly higher in *M. quadrifolia* compared with others. Among various MUFAs, oleic acid (C18:1n-9) was present in most of the plants and the amount was also higher compared with the others (Supplementary Tables 1A–C). MUFA content was also minimum in *P. stratiotes*. Though in small amounts two other monounsaturated fatty acids like, palmitoleic acid (C16:1n-9) and nervonic acid (C24:1), were present in all macrophytes, except *E. fluctuans* and *A. pinnata*. Another isomer of palmitoleic acid (C16:1n-7) was absent in two species of *Azolla* and *S. natans*.

**Table 1:** Saturated fatty acids (SFA) profiles of freshwater macrophytes cultured with organic manures (mg/100 g, dry weight).

**Macrophytes /Fatty acids**	**C14:0**	**C15:0**	**C16:0**	**C18:0**	**C20:0**	**C22:0**	**C24:0**	**∑SFA**
***Azolla microphylla***	6.55 ± 0.06^fg^	-	541.84 ± 18.38^de^	1.76 ± 0.89^h^	1.39 ± 0.23^h^	-	5.65 ± 0.23^ef^	**557** **±19.79**^**fg**^
***Azolla pinnata***	16.99 ± 0.69^e^	17.76 ± 8.72^a^	815.78 ± 3.39^b^	27.06 ± 0.36^cd^	4.08 ± 0.42e^f^	17.58 ± 1.45^a^	16.23 ± 1.09^a^	**915.51** **±8.08**^**b**^
***Enhydra fluctuans***	9.46 ± 0.29^f^	6.90 ± 2.29^bc^	377.47 ± 0.07^f^	29.34 ± 0.06^c^	2.01 ± 0.17g^h^	0.07 ± 0.03^d^	5.63 ± 1.02^ef^	**431.01** **±0.83**^**h**^
***Hydrilla verticillata***	20.03 ± 1.90^de^	4.40 ± 1.49^cde^	377.33 ± 16.67^f^	21.53 ± 1.68^ef^	17.91 ± 0.17^a^	0.89 ± 0.12^cd^	8.03 ± 0.57^de^	**450.14** **±5.075**^**h**^
***Ipomoea aquatica***	14.97 ± 0.73^ef^	3.64 ± 0.89^def^	622.79 ± 0.87^c^	68.69 ± 0.80^a^	4.54 ± 0.33^de^	0.07 ± 0.01^d^	14.47 ± 0.41^ab^	**729.22** **±1.90**^**d**^
***Lemna minor***	40.11 ± 0.05^a^	8.25 ± 0.13^b^	753.13 ± 0.05^b^	17.69 ± 0.06f^g^	5.65 ± 0.11^d^	5.04 ± 0.10^b^	12.81 ± 0.15^bc^	**842.69** **±0.55**^**c**^
***Marsilea quadrifolia***	14.50 ± 0.46^e^	1.92 ± 0.36^efg^	542.58 ± 6.39^de^	16.81 ± 0.47f^g^	1.38 ± 0.10^h^	0.84 ± 0.02^cd^	10.37 ± 0.81^cd^	**588.42** **±5.72**^**ef**^
***Pistia stratiotes***	5.29 ± 0.40^g^	3.85 ± 0.19^cde^	268.75 ± 0.06^g^	14.07 ± 1.16g	8.11 ± 0.10^c^	0.57 ± 0.04^cd^	10.10 ± 0.45^cd^	**310.77** **±0.70**^**i**^
***Salvinia molesta***	34.04 ± 5.04^b^	6.14 ± 0.85^bcd^	574.15 ± 19.84^cd^	23.35 ± 0.72^de^	2.99 ± 1.18^fg^	5.13 ± 0.02^b^	10.28 ± 0.48^cd^	**656.11** **±16.58**^**e**^
***Salvinia natans***	4.04 ± 0.37^g^	-	486.68 ± 2.72^e^	3.26 ± 0.07^h^	2.39 ± 0.18^gh^	-	5.08 ± 0.41^f^	**501.45** **±4.02**^**gh**^
***Spirodela polyrhiza***	24.26 ± 2.07^cd^	0.81 ± 0.04^fg^	509.94 ± 16.48^e^	24.84 ± 1.58^cde^	5.51 ± 0.62^d^	1.42 ± 0.03^cd^	7.85 ± 0.89^de^	**574.66** **±22.02**^**fg**^
***Wolffia globosa***	28.24 ± 0.60^c^	3.08 ± 0.29^def^	1084.31 ± 63.38^a^	54.25 ± 4.57^b^	11.31 ± 0.90^b^	2.07 ± 0.01^c^	10.87 ± 0.46^c^	**1194.14** **±71.13**^**a**^

*Values having the means (n = 3) in each row with different superscript are significantly (p < 0.05) different*.

**Table 2:** Monounsaturated fatty acids (MUFA) profiles of freshwater macrophytes cultured with organic manures (mg/100 g, dry weight).

**Macrophytes /Fatty acids**	**C16:1*n*-9**	**C16:1*n*-7**	**C17:1**	**C18:1*n*-9**	**C20:1*n*-9**	**C22:1*n*-9**	**C24:1**	**∑MUFA**
***Azolla microphylla***	9.85 ± 0.70^cde^	-	12.49 ± 0.75^c^	77.22 ± 1.20^e^	-	-	2.83 ± 0.74^b^	**102.39** **±1.91**^**e**^
***Azolla pinnata***	9.39 ± 0.52^cde^	-	91.52 ± 0.17^b^	106.75 ± 0.34^c^	-	-	-	**207.67** **±0.35**^**b**^
***Enhydra fluctuans***	-	25.29 ± 0.52^c^	-	44.99 ± 0.21^g^	0.28 ± 0.13^cd^	-	0.18 ± 0.13^c^	**70.75** **±0.73**^**f**^
***Hydrilla verticillata***	0.13 ± 0.02^g^	8.81 ± 0.42^d^	-	48.97 ± 1.60^g^	14.15 ± 0.14^a^	-	0.21 ± 0.03^c^	**72.28** **±3.67**^**f**^
***Ipomoea aquatica***	0.33 ± 0.02^f^	27.25 ± 0.29^c^	-	24.16 ± 1.20^h^	-	-	3.45 ± 0.62^b^	**55.20** **±1.73**^**g**^
***Lemna minor***	51.60 ± 0.08^a^	86.47 ± 0.15^a^	-	9.67 ± 0.10^i^	2.39 ± 0.04^b^	-	7.01 ± 0.11^a^	**157.17** **±0.12**^**c**^
***Marsilea quadrifolia***	7.86 ± 0.26^de^	24.61 ± 0.42^c^	168.24 ± 2.69^a^	127.93 ± 2.59^b^	0.54 ± 0.40^cd^	7.58 ± 0.76^b^	1.14 ± 0.74^c^	**337.94** **±7.36**^**a**^
***Pistia stratiotes***	0.65 ± 0.08^f^	10.59 ± 0.63^d^	-	30.66 ± 0.03^h^	0.25 ± 0.08^cd^	5.39 ± 0.25^c^	0.82 ± 0.03^c^	**48.38** **±0.14**^**g**^
***Salvinia molesta***	6.51 ± 0.89^e^	10.41 ± 2.38^d^	9.53 ± 0.49^d^	94.04 ± 0.02^d^	0.39 ± 0.05^cd^	3.63 ± 0.25^d^	0.51 ± 0.01^c^	**125.06** **±4.06**^**d**^
***Salvinia natans***	16.32 ± 0.74^b^	-	4.71 ± 0.34^e^	68.48 ± 1.18^f^	-	-	0.65 ± 0.01^c^	**90.16** **±0.28**^**e**^
***Spirodela polyrhiza***	13.81 ± 1.55^bc^	38.93 ± 2.70^b^	-	49.23 ± 5.09^g^	0.13 ± 0.02^cd^	3.30 ± 0.59^d^	0.06 ± 0.01^d^	**105.49** **±5.59**^**e**^
***Wolffia globosa***	12.00 ± 0.69^bcd^	40.67 ± 0.54^b^	-	137.22 ± 0.61^a^	0.64 ± 0.03^c^	17.65 ± 0.92^a^	0.20 ± 0.02^c^	**208.40** **±2.60**^**b**^

*Values having the means (n = 3) in each row with different superscript are significantly (p < 0.05) different*.

**Table 3:** Polyunsaturated fatty acids (PUFA) profiles of freshwater macrophytes cultured with organic manures (mg/100 g, dry weight).

**Macrophytes /Fatty acids**	**C18:2 *n*-6**	**C18:3 *n*-6**	**C20:2 *n*-6**	**C20:3 *n*-6**	**C20:4 *n*-6**	**∑*n*-6 PUFA**	**C18:3 *n*-3**	**∑*n*-3 PUFA**	***n*-3/n-6**
***Azolla microphylla***	96.72 ± 4.71^j^	-	-	-	16.71 ± 0.14^d^	**113.43** **±4.85**^**i**^	149.05 ± 4.98^i^	**149.05** **±4.98**^**i**^	1.31 ± 0.01^f^
***Azolla pinnata***	443.29 ± 1.13^c^	10.57 ± 0.31^b^	-	3.17 ± 0.62^de^	81.32 ± 0.42^a^	**538.36** **±2.49**^**c**^	591.33 ± 0.66^e^	**591.33** **±0.66**^**e**^	1.09 ± 0.01^h^
***Enhydra fluctuans***	329.01 ± 3.35^e^	-	1.11 ± 0.10^bc^	-	5.24 ± 0.24^e^	**335.38** **±3.01**^**e**^	608.37 ± 5.12^e^	**608.37** **±5.12**^**e**^	1.81 ± 0.01^d^
***Hydrilla verticillata***	168.35 ± 2.01^g^	-	0.97 ± 0.08^bc^	-	1.20 ± 0.17^g^	**170.53** **±1.89**^**g**^	405.91 ± 5.50^g^	**405.91** **±5.50**^**g**^	2.38 ± 0.06^b^
***Ipomoea aquatica***	384.86 ± 1.85^d^	-	0.95 ± 0.63^bc^	-	2.87 ± 0.09^f^	**388.69** **±1.31**^**d**^	1572.23 ± 2.87^a^	**1572.23** **±2.87**^**a**^	4.04 ± 0.01^a^
***Lemna minor***	601.47 ± 0.10^b^	-	3.35 ± 0.10^a^	5.33 ± 0.07^c^	-	**610.16** **±0.07**^**b**^	1505.63 ± 10.10^b^	**1505.63** **±10.10**^**b**^	2.46 ± 0.02^b^
***Marsilea quadrifolia***	255.25 ± 1.33^f^	-	0.91 ± 0.49^bc^	55.44 ± 0.55^a^	0.06 ± 0.01^i^	**311.68** **±2.31**^**f**^	473.56 ± 1.79^f^	**473.56** **±1.79**^**f**^	1.51 ± 0.01^e^
***Pistia stratiotes***	132.20 ± 0.67^h^	-	0.71 ± 0.04^c^	1.03 ± 0.03^f^	0.27 ± 0.01^h^	**134.22** **±2.11**^**h**^	322.97 ± 0.32^h^	**322.97** **±0.32**^**h**^	2.40 ± 0.03^b^
***Salvinia molesta***	131.23 ± 1.29^h^	-	2.39 ± 0.54^ab^	23.88 ± 1.30^b^	0.21 ± 0.01^h^	**157.72** **±0.56**^**g**^	97.09 ± 1.18^j^	**97.09** **±1.18**^**j**^	0.61 ± 0.01^j^
***Salvinia natans***	118.57 ±0.78^i^	-	-	-	19.21 ± 0.22^c^	**137.78** **±1.00**^**h**^	116.51 ± 0.89^j^	**116.51** **±0.89**^**j**^	0.85 ± 0.01^i^
***Spirodela polyrhiza***	368.28 ± 7.45^d^	-	1.00 ± 0.01^bc^	3.80 ± 0.34^cd^	0.93 ± 0.02^g^	**374.03** **±6.06**^**d**^	724.41 ± 12.66^d^	**724.41** **±12.66**^**d**^	1.93 ± 0.02^c^
***Wolffia globosa***	728.27 ± 17.19^a^	16.54 ± 1.18^a^	2.70 ± 0.05^a^	1.65 ± 0.01^ef^	22.44 ± 1.57^b^	**771.63** **±17.37**^**a**^	909.28 ± 16.17^c^	**909.28** **±16.17**^**c**^	1.17 ± 0.01^g^

*Values having the means (n = 3) in each row with different superscript are significantly (p < 0.05) different*.

The n-6 PUFA content was significantly higher in *W. globosa* compared with others. This was followed by *L. minor* and *A. pinnata*. The minimum level was found in *A. microphylla*. Among n-6 PUFA, LA (C18:2n-6) was the dominant one and was present in all macrophytes. Arachidonic acid (C20:4n-6) was the second dominant n-6 PUFA found in all macrophytes, except in *L. minor*. ALA (C18:3n-3) was the only member of n-3 PUFA present in all these macrophytes. ALA content was significantly higher in *I. aquatica* compared with others. This was followed by *L. minor* and *W. globosa*. The highest (4.04) n-3/n-6 was found in *I. aquatica* (Supplementary Table 2). The ratio ranged from 0.61 (*S. molesta*)−2.46 (*L. minor*) in other macrophytes.

## Discussion

A wide variation in the composition of freshwater macrophytes was recorded in the present study. The advantage of this study is that plants were cultured in the outdoor systems following a standard protocol (3). Therefore, almost the same quality of products is expected in a further study. There is scope for improvement in the nutritional value as the quality of the culture medium influences the composition of the plants.

In the present study, crude protein levels in three members of Lemnaceae family and *E. fluctuans* were above 30%, and protein contents of other macrophytes (except *P. stratiotes, S. natans*, and *M. quadrifolia*) were above 20%. The present study confirms the previous finding that macrophytes are rich sources of protein. The protein contents of *L. minor* and *S. polyrhiza* were 36.07 and 35.82%, respectively (3, 4). A previous study in Bangladesh reported that the protein contents of *E. fluctuans* and *I. aquatica* were 16.69 and 21.45%, respectively; macrophytes were collected from natural water bodies (15). In the present study, protein contents of *E. fluctuans* and *I. aquatica* were 16.35 and 7.51% higher compared with the same macrophytes studied in Bangladesh. Lipid contents of *I. aquatica, S. polyrhiza*, and *L. minor* ranged from 7.16 to 8.13% in the present study. The lipid contents of *E. fluctuans* and *I. aquatica* were 1.90 and 3.82% higher in the present study compared with the previous study (15). Ash contents of these two macrophytes were also higher in the present study compared with the previous one. Higher levels of ash contents of *H. verticillata, W. globosa*, and *P. stratiotes* compared with other macrophytes enhanced the nutritional value of these plants as feed ingredients for fish, poultry, and livestock. In the present study, lower levels of carbohydrates were observed in macrophytes compared with the plants harvested from the wild (15). Culture of macrophytes with organic manures enhanced the nutritional value of plants.

Among these macrophytes, highest levels of macrominerals, Na and Mg, were found in *P. stratiotes*. K and Ca were highest in *W. globosa* and *S. polyrhiza*, respectively. In the present study, among various macrophytes, *P. stratiotes* ranked second and fifth for Ca and K, respectively. A previous study reported the highest Ca level in *Hydrilla* sp., followed by *P. stratiotes* and *E. crassipes*. There was no variation in Mg level among these three macrophytes (16). Macromineral profile of leaves and roots of *P. stratiotes* collected from a natural water body of Nigeria was documented (17). This study showed that Na, K, Ca, and Mg contents were 3.73, 32.83, 2.30, and 3.70 g/kg of leaves, respectively. In the present study, Na, Ca, and Mg contents were 47, 20, and 30%, respectively, higher in *P. stratiotes* compared with the plants studied in Nigeria. K content was almost the same in the plants grown in two different conditions. The Na, Ca, Zn, and Cu contents were higher in *I. aquatica* grown in Bangladesh compared with the macrophytes assayed in the present study (15). Although, Mg, K, and Fe contents were higher in the *I. aquatica* assayed in the present study compared with the plants studied in Bangladesh, Na, Mg, and K contents were higher in *E. fluctuans* evaluated in the present study compared with the previous study in Bangladesh. The Na, K, Mg, and Ca contents were higher in *A. filiculoides* and *S. molesta* grown in swine lagoons compared with the present study (18). In *A. filiculoides*, Na, K, Mg, and Ca contents were 2.77, 22.5, 5.04, and 9.3 g/kg (dry matter), respectively. In *S. molesta* Na, K, Mg, and Ca contents were 4.44, 34.7, 5.18 and 10.6 g/kg (dry matter), respectively.

In the present study, Na to K ratio ranged from 0.038 (*M. quadrifolia*) −0.276 (*I. aquatica*). The ratio was 0.063, 0.109, 0.121, 0.160, 0.177, 0.182, 0.185, 0.205, 0.218, and 0.238 in *L. minor, W. globosa, S. polyrhiza, A. pinnata, E. fluctuans, H. verticillata, A. microphylla, S. molesta, P. stratiotes*, and *S. natans*, respectively. In all these macrophytes, the ratio of Na to K is less than the WHO/FAO-recommended ratio for an adult human, i. e., <0.49 (19). Various studies showed the effect of culture medium on the mineral profile of macrophytes (8, 20, 21). In different species of duckweeds Na: K varied from 0.027–1.49 (K: Na = 0.67–37). In *Wolffia*, the ratio was 0.025 (K: Na = 40) and in another species, *W. microscopica* it was 0.003 (K: Na = 276).

In the present study, the Mg: Ca varied from 1.20 (*L. minor*)−4.65 in (*E. fluctuans*). Ca has been serving as the main structural mineral and helps in metabolism. It serves as a signal for vital physiological processes. Mg, the fourth most abundant cation in the body, is a co-factor for 350 cellular enzymes, most of which are involved in energy metabolism (22), hence, the ratio of Mg: Ca should be maintained. The Mg: Ca ratio was 0.4 in duckweed (21) and 0.5 in other species, *W. microscopica* (8). In the present study, the ratio was 1.28 for *W. globosa*.

The trace minerals analysis showed that among these macrophytes, *P. stratiotes* was a rich source for Mo, Fe, and Al. This macrophyte also has considerable amounts of Mn, Zn, and Sr. *A. microphylla* and *A. pinnata* were also rich sources of Fe and Mo. In a different strain of *W. arrhiza*, Fe contents ranged from 0.16–0.29 μg/g freeze-dried sample (23). The Fe content of *W. globosa* was 254.12 μg/g in the present study. Higher levels of Zn and Cu were found in *I. aquatica* grown in Bangladesh compared with the present study; Fe content was higher in the present study compared with the previous one (15). Fe content of *E. fluctuans* grown in two different environments was the same. Zn and Cu contents were lower in the plants assayed in the present study compared with the plants studied in Bangladesh. The Cu content of *S. molesta* grown in swine lagoons was 13 g/kg, dry weight (18). In the present study, Cu content of *S. molesta* was less compared with the previous study.

In the present study, the highest level of ultra-trace mineral Se was found in *S. natans*. This important mineral was also present in *H. verticillata* and *P. stratiotes*. It was interesting to record that Se was absent in *S. molesta, S. polyrhiza, E. fluctuans*, and *I. aquatica*. The Se content of freeze-dried *W. arrhiza* was <0.03 μg/g (23). In the present study, Se content of *W. globosa* was higher compared with the previous study. Significantly higher Cr levels were found in *P. stratiotes, A. microphylla*, and *A. pinnata* compared with other macrophytes. A significantly higher Co level was found in *H. verticillata* compared with the others. This macrophyte was followed by *P. stratiotes and S. natans*. Co content in all these macrophytes was >0.50 μg/g (dry weight). Among these macrophytes, the highest Ni content was found in *S. polyrhiza*, and this macrophyte was followed by *H. verticillata* and *P. stratiotes*. In the present study, the contents of heavy metals *viz*. Cd, Cu, Pb, and Sn of macrophytes were within the permissible limits (Cd: 0.2, Cu: 73.3, Pb: 0.3, Sn: 250 Zn: 99.40; mg/kg of wet weight) of WHO/FAO (24). In the present study, the mineral composition was evaluated in the dry sample. Therefore, the moisture (minimum 90%) contents of the samples should be considered at the time of comparison with the permissible limit of WHO/FAO for humans (where fresh plants were considered). In seaweeds, there is no regulation on the maximum heavy metals contents (25).

Various studies showed the dietary requirements of different macro, trace, and ultra-trace minerals for different animals (Supplementary Tables 3A,B). Na requirements of grass carp (*Ctenopharyngodon idella*), poultry, cattle, and humans are 2, 0.012–0.200, 0.96 g/kg diet, and 2.4 g/day, respectively. Among various fishes and prawns (*Pinneaus indicus*), Mg requirements vary from 0.4 to 0.946 g/kg of diets. K requirements recorded for common carp (*Cyprinus carpio*), grass carp, and Nile tilapia (*Orechromis niloticus*) are as follows: 0.9–12.4, 4.6, and 2.1–3.3 g/kg diets. K requirements for poultry, cattle, and humans are 0.3 and 2.4 g/kg diet and 3.5 g/day, respectively. Among different groups of fishes, rohu (*Labeo rohita*), common carp, grass carp, catla (*Catla catla*), and Nile tilapia require 1.9, 0.1, 2, 1.9 and 7 g Ca/kg diet, respectively. Ca requirements for poultry, cattle, and humans are 8 and 5.12 g/kg diet and 1.0 g/day, respectively. Among various fishes, Mn, Fe, Zn, and Co requirements vary from 12–25, 30–200, 15–79, and 0.01–0.5 mg/kg diet, respectively. Nile tilapia requires Se and Cr at the rate of 0.4 and 139.6 mg/kg diet, respectively. Fe, Zn, Cu, Se, Cr, and Co requirements are also evaluated for poultry, cattle, and humans. In channel catfish *Ictaluraus punctatus*, Fe, Cu, Mn, Zn, Se, and Co requirements were 30, 5, 25, 200, 0.1, and 0.05 mg/kg feed, respectively (26). In fish, Fe deficiency causes hypochromic microcytic anemia, Co and Mn deficiencies result in poor growth; Zn deficiency causes growth depression, cataract, and caudal fin and skin erosion; Se deficiency results in muscular dystrophy. In fish nutrition, Co plays a significant role. In common carp, the addition of cobalt chloride/cobalt nitrate enhanced the growth and hemoglobin formation (27). Therefore, supplementation of freshwater macrophytes may help to overcome the mineral deficiency in fish and other animals without showing any negative impact (9).

The fatty acid compositions of the two duckweeds *L. minor* and *S. polyrhiza* showed similarity with the previous study (3, 4). In the present study, palmitic acid and oleic acid were the dominant SFA and MUFA, respectively. Similar results were also found in four duckweeds, *Landoltia, Lemna, Wolffiella*, and *Wolffia* (8). The fatty acid compositions of four aquatic plants *S. cuculata, Trapa natans, L. minor*, and *I. reptans* showed that cis-15 tetracosenoic acid and 9-hexadecenoic acid were the dominant fatty acids, and highly unsaturated fatty acids contents were higher compared with the saturated fatty acids (28). In the present study, LA was the major contributor for n-6 PUFA in all plants, and except in *L. minor*, arachidonic acid was also found in all macrophytes. ALA was the only member of n-3 PUFA present in these macrophytes. The presence of LA and ALA were recorded in duckweeds (8). The freshwater teleosts are capable of converting ALA to long-chain polyunsaturated fatty acids (LC-PUFA) like eicosapentaenoic acid (EPA; 20:5*n*-3) and docosahexaenoic acid (DHA; 22:6n-3) (29–31). Therefore, the feeding of fish with freshwater macrophytes-based diets helps to fulfill the LC-PUFA requirements of cultured fish (32, 33). The n-6/n-3 PUFA was always <1; it ranged from 0.48–0.94 in different *Wolffia* species (23). A similar result was also found in the present study, except in two species of *Salvania*, where the ratio was >1.0.

## Conclusion

Among these macrophytes, Na, Mg, Cr, and Fe contents were maximum in *P. stratiotes*; this macrophyte ranked second for Co, Sr, and Ca. *H. verticillata* was the richest source for Cu, Mn, Co, and Zn, and it ranked second for Se. Ca, Mg, Sr, and Ni contents were higher in *S. polyrhiza* compared with the others. *S. natans* and *W. globosa* were rich sources for Se and K, respectively. All these macrophytes were rich sources of n-6 and n-3 fatty acids. This study shows that macrophytes have an immense potential to be used as rich sources of minerals, as well as n-6 and n-3 PUFA for fish, poultry, and livestock.

## Data Availability Statement

The original contributions presented in the study are included in the article/Supplementary Material, further inquiries can be directed to the corresponding author/s.

## Author Contributions

JS, RC, and DRT designed the study. GK, RG, AS, NK, JS, and RC grown the macrophytes and analyzed samples. RC, DRT, GK, and JS wrote the manuscript. GK, AS, RG, NK, RC, DRT, and JS prepared tables. All authors contributed to the article and approved the submitted version.

## Funding

This study has been supported by the Department of Biotechnology, DBT, New Delhi, India (Dy. No.102/IFD/SAN/4678/2015-2016) and Biotechnology and Biological Science Research Council, BBSRC (BB/N005031/1). GK is thankful to the Indian Council of Medical Research (3/1/3/JRF-2017/HRD-LS/55340/01).

## Conflict of Interest

The authors declare that the research was conducted in the absence of any commercial or financial relationships that could be construed as a potential conflict of interest.

## Publisher's Note

All claims expressed in this article are solely those of the authors and do not necessarily represent those of their affiliated organizations, or those of the publisher, the editors and the reviewers. Any product that may be evaluated in this article, or claim that may be made by its manufacturer, is not guaranteed or endorsed by the publisher.
