# Supplementary material for: Freshwater Macrophytes: A Potential Source of Minerals and Fatty Acids for Fish, Poultry, and Livestock
Source: Front Nutr. 2022 Apr 11;9:869425. doi: 10.3389/fnut.2022.869425 (PMC9036174; doi:10.3389/fnut.2022.869425)
Supplement: Supplementary file 1 [file Table_1.docx]

**SUPPLEMENTARY TABLE 1(A) |** Saturated fatty acids (SFA) contents of freshwater macrophytes (Area %).

| Macrophytes / Fatty acids | C14:0 | | C15:0 | C16:0 | C18:0 | C20:0 | C22:0 | C24:0 | | ∑SFA |
| --- | --- | --- | --- | --- | --- | --- | --- | --- | --- | --- |
| *Azolla microphylla* | 0.71 ± 0.02 | - | | 58.76 ± 0.02 | 0.19 ± 0.01 | 0.15 ± 0.02 | - | 0.61 ± 0.01 | 60.43 ± 0.08 | |
| *Azolla pinnata* | 0.75 ± 0.03 | 0.79 ± 0.03 | | 36.21 ± 0.06 | 1.20 ± 0.02 | 0.18 ± 0.02 | 0.78 ± 0.07 | 0.72 ± 0.05 | 40.64 ± 0.25 | |
| *Enhydra fluctuans* | 0.65 ± 0.02 | 0.48 ± 0.06 | | 26.11 ± 0.15 | 2.04 ± 0.01 | 0.14 ± 0.01 | 0.01 ± 0.00 | 0.39 ± 0.07 | 29.82 ± 0.22 | |
| *Hydrilla verticillata* | 1.82 ± 0.09 | 0.40 ± 0.04 | | 34.36 ± 1.87 | 1.96 ± 0.17 | 3.16 ± 0.02 | 0.08 ± 0.01 | 0.73 ± 0.07 | 42.52 ± 2.43 | |
| *Ipomoea aquatica* | 0.55 ± 0.03 | 0.13 ± 0.01 | | 22.69 ± 0.03 | 2.50 ± 0.04 | 0.17 ± 0.01 | 0.01 ± 0.00 | 0.53 ± 0.01 | 26.56 ± 0.20 | |
| *Lemna minor* | 1.28 ± 0.01 | 0.27 ± 0.02 | | 24.03 ± 0.08 | 0.57 ± 0.01 | 0.18 ± 0.02 | 0.16 ± 0.01 | 0.41 ± 0.04 | 26.91 ± 0.09 | |
| *Marsilea quadrifolia* | 0.85 ± 0.03 | 0.11 ± 0.02 | | 31.77 ± 0.12 | 0.98 ± 0.02 | 0.08 ± 0.01 | 0.05 ± 0.01 | 0.61 ± 0.05 | 34.45 ± 0.06 | |
| *Pistia stratiotes* | 0.65 ± 0.05 | 0.47 ± 0.02 | | 32.92 ± 0.07 | 1.72 ± 0.15 | 0.99 ± 0.01 | 0.07 ± 0.01 | 1.24 ± 0.06 | 38.07 ± 0.17 | |
| *Salvinia molesta* | 3.29 ± 0.53 | 0.59 ± 0.07 | | 55.41 ± 1.21 | 2.25 ± 0.04 | 0.29 ± 0.03 | 0.50 ± 0.01 | 0.99 ± 0.03 | 63.32 ± 0.80 | |
| *Salvinia natans* | 0.48 ± 0.04 | - | | 57.53 ± 0.16 | 0.39 ± 0.13 | 0.28 ± 0.02 | - | 0.60 ± 0.05 | 59.28 ± 0.31 | |
| *Spirodela polyrhiza* | 1.36 ± 0.08 | 0.05 ± 0.01 | | 28.67 ± 0.18 | 1.40 ± 0.05 | 0.31 ± 0.03 | 0.08 ± 0.01 | 0.44 ± 0.01 | 32.30 ± 0.40 | |
| *Wolffia globosa* | 0.92 ± 0.05 | 0.10 ± 0.01 | | 35.14 ± 0.83 | 1.76 ± 0.09 | 0.37 ± 0.02 | 0.07 ± 0.01 | 0.35 ± 0.01 | 38.69 ± 0.96 | |

**SUPPLEMENTARY TABLE 1(B) |** Monounsaturated fatty **acids** (MUFA) contents of freshwater macrophytes (Area %).

| Macrophytes / Fatty acids | C16:1n-9 | C16:1n-7 | C17:1 | C18:1n-9 | C20:1n-9 | C22:1n-9 | C24:1 | ∑MUFA |
| --- | --- | --- | --- | --- | --- | --- | --- | --- |
| *Azolla microphylla* | 1.07 ± 0.04 | - | 1.35 ± 0.04 | 8.38 ± 0.16 | - | - | 0.31 ± 0.01 | 11.11 ± 0.17 |
| *Azolla pinnata* | 0.42 ± 0.02 | - | 4.06 ± 0.02 | 4.74 ± 0.01 | - | - | - | 9.22 ± 0.04 |
| *Enhydra fluctuans* | 0.02 ± 0.00 | 1.75 ± 0.03 | - | 3.11 ± 0.01 | - | - | 0.01 ± 0.00 | 4.91 ± 0.02 |
| *Hydrilla verticillata* | 0.01 ± 0.00 | 0.80 ± 0.08 | - | 4.46 ± 0.23 | 2.50 ± 0.04 | - | 0.02 ± 0.00 | 7.79 ± 0.14 |
| *Ipomoea aquatica* | 0.01±0.00 | 0.99 ± 0.01 | - | 0.88 ± 0.04 | - | - | 0.13 ± 0.02 | 2.01 ± 0.06 |
| *Lemna minor* | 1.65 ± 0.01 | 2.75 ± 0.01 | - | 0.31 ± 0.01 | 0.08 ± 0.01 | - | 0.23 ± 0.02 | 5.02 ± 0.02 |
| *Marsilea quadrifolia* | 0.24 ± 0.04 | 1.44 ± 0.01 | 9.85 ± 0.08 | 7.49 ± 0.09 | 0.03 ± 0.00 | 0.44 ± 0.04 | 0.07 ± 0.01 | 19.57 ± 0.06 |
| *Pistia stratiotes* | 0.08 ± 0.01 | 1.30 ± 0.02 | - | 3.76 ± 0.01 | 0.03 ± 0.00 | 0.66 ± 0.03 | 0.10 ± 0.01 | 5.93 ± 0.04 |
| *Salvinia molesta* | 0.63 ± 0.09 | 1.01 ± 0.04 | 0.92 ± 0.06 | 9.08 ± 0.11 | 0.04 ± 0.01 | 0.35 ± 0.03 | 0.05 ± 0.01 | 12.08 ± 0.55 |
| *Salvinia natans* | 1.93 ± 0.09 | - | 0.56 ± 0.04 | 8.09 ± 0.12 | - | - | 0.08 ± 0.01 | 10.66 ± 0.02 |
| *Spirodela polyrhiza* | 0.78 ± 0.11 | 2.19 ± 0.10 | - | 2.76 ± 0.21 | 0.01 ± 0.00 | 0.19 ± 0.04 | - | 5.93 ± 0.16 |
| *Wolffia globosa* | 0.40 ± 0.07 | 1.32 ± 0.06 | - | 4.45 ± 0.06 | 0.02 ±0.00 | 0.57 ± 0.01 | 0.01 ± 0.00 | 6.76 ± 0.15 |

**SUPPLEMENTARY TABLE 1(C) |** Polyunsaturated fatty **acids** (PUFA) contents of freshwater macrophytes (Area %).

| Macrophytes / Fatty acids | C18:2 n-6 | C18:3 n-6 | C20:2 n-6 | C20:3 n-6 | C20:4 n-6 | ∑n-6 PUFA | C18:3 n-3 | ∑ n-3 PUFA | n-3/n-6 |
| --- | --- | --- | --- | --- | --- | --- | --- | --- | --- |
| *Azolla microphylla* | 10.49 ± 0.15 | - | - | - | 1.83 ± 0.06 | 12.31 ± 0.09 | 16.17 ± 0.01 | 16.17 ± 0.01 | 1.31 ± 0.01 |
| *Azolla pinnata* | 19.68 ± 0.10 | 0.47 ± 0.01 | - | 0.14 ± 0.03 | 3.61 ± 0.03 | 23.90 ± 0.17 | 26.25 ± 0.04 | 26.25 ± 0.04 | 1.10 ± 0.01 |
| *Enhydra fluctuans* | 22.76 ± 0.11 | - | 0.08 ± 0.01 | - | 24.45 ± 0.02 | 23.25 ± 0.03 | 42.08 ± 0.12 | 42.08 ± 0.12 | 1.81 ± 0.01 |
| *Hydrilla verticillata* | 15.32 ± 0.27 | - | 0.09 ± 0.01 | - | 0.11 ± 0.01 | 15.52 ± 0.33 | 36.94 ± 0.12 | 36.94 ± 0.12 | 2.38 ± 0.06 |
| *Ipomoea aquatica* | 14.02 ± 0.03 | - | 0.03 ± 0.00 | - | 0.10 ± 0.01 | 14.16 ± 0.01 | 57.27 ± 0.06 | 57.27 ± 0.06 | 4.04 ± 0.01 |
| *Lemna minor* | 19.19 ± 0.06 | - | 0.11 ± 0.01 | 0.17 ± 0.02 | - | 19.47 ± 0.06 | 48.04 ± 0.17 | 48.04 ± 0.17 | 2.47 ± 0.02 |
| *Marsilea quadrifolia* | 14.95 ± 0.04 | - | 0.05 ± 0.00 | 3.25 ± 0.01 | - | 18.25 ± 0.01 | 27.73 ± 0.11 | 27.73 ± 0.11 | 1.52 ± 0.01 |
| *Pistia stratiotes* | 16.19 ± 0.05 | - | 0.09 ± 0.01 | 0.13 ± 0.01 | 0.03 ± 0.00 | 16.44 ± 0.22 | 39.56 ± 0.05 | 39.56 ± 0.05 | 2.41 ± 0.04 |
| *Salvinia molesta* | 12.67 ± 0.04 | - | 0.23 ± 0.06 | 2.31 ± 0.05 | 0.02 ± 0.00 | 15.23 ± 0.25 | 9.37 ± 0.01 | 9.37 ± 0.01 | 0.62 ± 0.01 |
| *Salvinia natans* | 14.02 ± 0.13 | - | - | - | 2.27 ± 0.03 | 16.29 ± 0.16 | 13.77 ± 0.14 | 13.77 ± 0.14 | 0.85 ± 0.01 |
| *Spirodela polyrhiza* | 20.71 ± 0.12 | - | 0.06 ± 0.01 | 0.22 ± 0.02 | 0.05 ± 0.00 | 21.03 ± 0.21 | 40.74 ± 0.35 | 40.74 ± 0.35 | 1.94 ± 0.08 |
| *Wolffia globosa* | 23.63 ± 0.26 | 0.54 ± 0.02 | 0.09 ± 0.01 | 0.05 ± 0.00 | 0.73 ± 0.08 | 25.04 ± 0.31 | 29.51 ± 0.50 | 29.51 ± 0.50 | 1.18 ± 0.01 |

**SUPPLEMENTARY TABLE 2 |** The n-3/n-6 and n-6/n-3 of **freshwater** macrophytes (Area %).

| Macrophytes/Fatty Acids | ∑ SFA % | ∑ MUFA % | ∑n-6 PUFA % | | ∑n-3 PUFA % | n-3/n-6 | n-6/ n-3 |
| --- | --- | --- | --- | --- | --- | --- | --- |
| *Azolla microphylla* | 60.43 ± 0.08 | 11.11 ± 0.17 | | 12.31 ± 0.09 | 16.17 ± 0.01 | 1.31 ± 0.01 | 0.76 ± 0.01 |
| *Azolla pinnata* | 40.64 ± 0.25 | 9.22 ± 0.04 | | 23.90 ± 0.17 | 26.25 ± 0.04 | 1.10 ± 0.01 | 0.91 ± 0.02 |
| *Enhydra fluctuans* | 29.82 ± 0.22 | 4.91 ±0.02 | | 23.25 ± 0.03 | 42.08 ± 0.12 | 1.81 ± 0.01 | 0.55 ± 0.01 |
| *Hydrilla verticillata* | 42.52 ± 2.43 | 7.79 ± 0.14 | | 15.52 ± 0.33 | 36.94 ± 0.12 | 2.38 ±0.06 | 0.42 ± 0.01 |
| *Ipomoea aquatica* | 26.56 ± 0.12 | 2.01 ± 0.01 | | 14.16 ± 0.01 | 57.27 ± 0.06 | 4.04 ± 0.01 | 0.25 ± 0.01 |
| *Lemna minor* | 26.91 ± 0.09 | 5.02 ± 0.02 | | 19.47 ± 0.06 | 48.04 ± 0.17 | 2.47 ± 0.02 | 0.41 ±.03 |
| *Marsilea quadrifolia* | 34.45 ± 0.06 | 19.57 ± 0.06 | | 18.25 ± 0.01 | 27.73 ± 0.11 | 1.52 ± 0.01 | 0.66 ± 0.04 |
| *Pistia stratiotes* | 38.07 ± 0.17 | 5.93 ± 0.04 | | 16.44 ± 0.22 | 39.56 ± 0.05 | 2.41 ± 0.04 | 0.42 ± 0.01 |
| *Salvinia molesta* | 63.32 ± 0.80 | 12.08 ± 0.55 | | 15.23 ± 0.25 | 9.37 ± 0.01 | 0.62 ± 0.01 | 1.62 ± 0.05 |
| *Salvinia natans* | 59.28 ± 0.31 | 10.66 ± 0.02 | | 16.29 ± 0.16 | 13.77 ± 0.14 | 0.85 ± 0.01 | 1.18 ± 0.03 |
| *Spirodela polyrhiza* | 32.30 ± 0.40 | 5.93 ± 0.16 | | 21.03 ± 0.21 | 40.74 ± 0.35 | 1.94 ± 0.04 | 0.52 ± 0.02 |
| *Wolffia globosa* | 38.69 ± 0.96 | 6.76 ± 0.15 | | 25.04 ± 0.31 | 29.51 ± 0.50 | 1.18 ± 0.01 | 0.85 ± 0.11 |

| Minerals | Prawn | Common carp | Rohu | Catla | Grass carp | Nile tilapia | Sea bass |
| --- | --- | --- | --- | --- | --- | --- | --- |
| Macrominerals (μg/g) | | | | | | | |
| Sodium (Na) | 0.003 | - | - | - | 2000 | - | - |
| Magnesium (Mg) | 946 | 400 – 500 | - | 600 | 300 | 600 – 800 | - |
| Potassium (K) | 0.04 | - | - | - | 4600 | 2100 – 3300 | - |
| Calcium (Ca) | 320 | - | 1900 | 1900 | 2000 | 7000 | - |
| Trace minerals (μg/g) | | | | | | | |
| Manganese (Mn) | 30 | 13 | - | 13 | 13 – 20 | 12 | 25 |
| Aluminium (Al) | 0.02 | - | - | - | - | - | - |
| Iron (Fe) | 133.5 | 150 | 30 – 170 | 150 | 200 | 60 | 30 |
| Zinc (Zn) | 165 | 15 – 30 | 15 – 40 | 15 – 30 | 34 – 40 | 30 – 79 | 50 |
| Copper (Cu) | 51 | 3 | - | 3 | 3.2 – 4 | 2 – 3 | 3 |
| Ultra-trace minerals (μg/g) | | | | | | | |
| Selenium (Se) | 75 | - | - | - | - | 0.4 | 0.1 |
| Chromium (Cr) | - | - | - | - | - | 139.6 | 0.25 |
| Cobalt (Co) | 2.1 | 0.1 | 0.05 – 1.0 | - | 0.12 | - | 0.5 |

**SUPPLEMENTARY TABLE 3 (A) |** Recommended dietary requirements of minerals for prawn, common carp, rohu, catla, Nile tilapia, grass carp and sea bass (μg/g of diet) (34, 35).

**SUPPLEMENTARY TABLE 3 (B) |** Recommended dietary requirements of minerals for poultry, cattle (units/g of diet) and humans (units/day).

| Minerals | Poultry^36^ | Cattle^37^ | Human^38^ |
| --- | --- | --- | --- |
| Macrominerals | | | |
| Sodium (Na) | 0.012 - 0.200 mg | 0.96 mg | 2300 mg |
| Magnesium (Mg) | - | 0.79 mg | 420 mg |
| Potassium (K) | 0.300 mg | 2.4 mg | 4700 mg |
| Calcium (Ca) | 8 mg | 5.12 mg | 1300 mg |
| Trace minerals | | | |
| Molybdenum (Mo) | - | - | 45 μg |
| Manganese (Mn) | 60 μg | 9.59 μg | 2300 μg |
| Iron (Fe) | 80 μg | 218 μg | 18000 μg |
| Zinc (Zn) | 40 μg | 61 μg | 11000 μg |
| Copper (Cu) | 8 μg | 9.53 μg | 900 μg |
| Ultra-trace minerals | | | |
| Selenium (Se) | 0.15 μg | 0.57 μg | 55 μg |
| Chromium (Cr) | - | 2.53 μg | 35 μg |
| Cobalt (Co) | - | 2.78 μg | 5-8 μg^39^ |

,
